# Prediction of fluid responsiveness in mechanically ventilated patients in surgical intensive care unit by pleth variability index and inferior vena cava diameter

**DOI:** 10.1186/s42077-020-00097-4

**Published:** 2020-10-07

**Authors:** Diaaeldin Badr Metwally Kotb Aboelnile, Mohamed Ismail Abdelfattah Elseidy, Yasir Ahmed Elbasiony Mohamed Kenawey, Ibrahim Mohammed Alsayed Ahmed Elsherif

**Affiliations:** Department of Anesthesiology, Intensive care and Pain Management, Faculty of Medicine, Ain-Shams University, Abbassia, Cairo, 11591 Egypt

**Keywords:** Fluid responsiveness, Hemodynamic monitoring, Inferior vena cava diameter, Pleth variability index, Ultrasound

## Abstract

**Background:**

Patients may have signs of hypovolemia, but fluid administration is not always beneficial. We are in need of bedside devices and techniques, which can predict fluid responsiveness effectively and safely. This study is aiming to compare the effectiveness and reliability of the pleth variability index (PVI) and IVC distensibility index (dIVC) as predictors of fluid responsiveness by simultaneous recordings in all sedated mechanically ventilated patients in the surgical intensive care unit (ICU). We used the passive leg raising test (PLR) as a harmless reversible technique for fluid challenge, and patients were considered responders if the cardiac index (CI) measured by transthoracic echocardiography (TTE) increased ≥ 15% after passive leg raising test (PLR).

**Results:**

This observational cross-sectional study was performed randomly on 88 intubated ventilated sedated patients. Compared with CI measured by transthoracic echocardiography, the dIVC provided 79.17% sensitivity and 80% specificity at a threshold value of > 19.42% for fluid responsiveness prediction and was statistically significant (*P* < .0001), with an area under the curve (AUC) of 0.886 (0.801–0.944), while PVI at a threshold value of > 14% provided 93.75% sensitivity and 87.5% specificity and was statistically significant (*P* < .0001), with an AUC of 0.969 (0.889–0.988).

**Conclusion:**

PVI and dIVC are effective non-invasive bedside methods for the assessment of fluid responsiveness in ICU for intubated ventilated sedated patients with sinus rhythm, but PVI has the advantage of being continuous, operator-independent, and more reliable than dIVC.

## Background

Perioperative prediction of fluid responsiveness has been a challenge for many years. It is known as the ability of the circulation to increase cardiac output (CO) in response to volume expansion. Accommodation of the large volume of venous return (VR) is done by stretching ventricles, which is known as cardiac preload. Since preload is related to CO, increased negativity of intrathoracic pressure (ITP) during inspiration subsequently increases VR and subsequently CO, and the reverse occurs during expiration (Chu et al., 2016).

Hemodynamic optimization by intravenous (IV) fluid administration is very important to correct any fluid deficits created by fasting, blood loss, and urinary excretion, or in septic and other critically ill patients to improve oxygen delivery and overall hemodynamic function. However, it may be ineffective or harmful to patients if no suitable monitoring methods are used. Interstitial fluid accumulation by more volume expansion may worsen oxygen diffusion to the tissues and decrease myocardial compliance. Therefore, it became essential to develop an approach for the evaluation of patients who are likely to get benefit from fluid administration (Cumpstey et al., 2016).

Methods for assessment of volume responsiveness depending on what and how surrogate is measuring can be classified into static and dynamic measures (Jalil & Cavallazzi, 2018).

Swan and Ganz developed in 1970 the pulmonary artery flow-directed catheter. It has the ability to measure the pulmonary artery occlusion pressure (PAOP), but due to its complications and the same limitations as central venous pressure (CVP) (Chatterjee, 2009; Marik & Cavallazzi, 2013), multiple studies found that it cannot be a good predictor of fluid responsiveness (Marik & Lemson, 2014), as these static techniques cannot predict the effect on ITP during inspiration and expiration (Wise et al., 2017).

Michard and others discovered that the interaction between the heart and lung during mechanical ventilation could be used for the prediction of fluid responsiveness (Michard et al., 2000). Based on this idea, dynamic measurements such as pulse pressure variation (PPV) and stroke volume variation (SVV) were used to predict fluid responsiveness in a more accurate way, especially in sedated mechanically ventilated patients, but due to being invasive and affected by multiple clinical factors (Marik & Lemson, 2014), non-invasive bedside and continuous techniques became more popular (Haas et al., 2012).

Pleth variability index (PVI) is a dynamic variable, which has recently gained a lot of interest. By using the amplitude of the pulse oximeter waveform, it measures the respiratory variation continuously and automatically. Therefore, it became an effective dynamic fluid response predictor in sedated mechanically ventilated patients with sinus rhythm by providing simple numeric value on the monitor screen (Chu et al., 2016).

Ultrasound calculation of inferior vena cava distensibility index (dIVC) in mechanically ventilated patients has been reported as a widely available and non-invasive measure for prediction of fluid responsiveness in multiple patient settings (De Backer & Fagnoul, 2014). It can also be used with an ultrasound of the lungs and heart to give a comprehensive sonographic picture especially for critically ill patients in ICU (Evans et al., 2014).

We aim to compare the accuracy and reliability of the PVI and dIVC as predictors of fluid responsiveness by simultaneous recordings in mechanically ventilated sedated patients in our surgical ICU.

## Methods

After approval of the ethical committee in the Faculty of Medicine, number FMASU M D 89/2018, this observational prospective study was conducted over 88 patients for 1 year from March 2018 to March 2019. Written informed consent was obtained from the patients’ legal guardians after explaining the procedure and its potential complications.

### Inclusion criteria

The inclusion criteria are age ≥ 18 years, admission to the ICU preoperative (in need of ventilation) or postoperative, and sedated on controlled mechanical ventilation with arterial and central venous catheters. The included patients were in need of IV fluid challenge for resuscitation based on the clinical characteristics (systolic arterial blood pressure (BP) < 90 mmHg, or mean arterial pressure (MAP) < 65 mmHg, or a fall of > 20 mmHg from the baseline of MAP, or with signs of hypoperfusion as oliguria less than 0.5 ml/kg/h and arterial lactate > 2.5 mmol/L).

### Exclusion criteria

The exclusion criteria are spontaneous breathing, poor cardiac echogenicity, cardiac arrhythmia, severe valvular heart disease or intracardiac shunt, impaired left ventricular function (ejection fraction < 40%), ascites, pregnancy, and any contraindication to fluid resuscitation, such as congestive heart failure, evidence of fluid overload, and renal dysfunction.

All patients were fully assessed with general physical examination, and their baseline characteristics, including age, gender, height, weight, body mass index, body surface area, and Acute Physiology and Chronic Health Evaluation score (APACHE) II score (Knaus et al., 1985), were recorded.

Patients were sedated using IV titration of fentanyl starting with 0.5 mcg/kg and midazolam starting with 0.5–1 mg to achieve a Ramsay sedation score of 4–6 (Ramsay et al., 1974), before muscle relaxation with 0.6 mg/kg rocuronium IV. Patients were mechanically ventilated (Newport™ e360 Ventilator; Newport Medical Instrument, CA) in volume control mode with tidal volume = 8 ml/kg of predicted body weight, inspiratory to expiratory ratio = 1:2, and positive end-expiratory pressure = 5 cm/H_2_O, and end-tidal carbon dioxide was monitored and maintained between 30 and 35 mmHg by adjusting the respiratory rate.

Recorded measurements were heart rate (HR), MAP, CVP with a zero referenced to the middle axillary line, PVI, dIVC, and cardiac index (CI). At first, every patient was positioned semi-recumbent with an angle of 45° between the trunk and the bed plane to record the initial measurements. Then, in the passive leg raising (PLR) position, other measurements were recorded after 1 min from leg elevation to 45° with the horizontal plane of the trunk. Positioning was done by an automatic bed mechanism controlled by a remote controller.

CO was measured by the same operator using transthoracic echocardiography (TTE) (ACUSON X300™ Ultrasound System, Premium Edition, Siemens Healthcare, Mountain View), as the product of stroke volume (SV) and heart rate (HR). SV is equal to the product of the aortic cross-sectional area, and the distance of a column of blood travels through it with each stroke, which is known as the velocity time integral (VTI) (Desai & Garry, 2018).

The cross-sectional area of the aortic annulus was estimated from the diameter (*D*) of the annulus using the parasternal long-axis view of the left ventricle. From this view, *D* is the maximal distance between the hinge points of the anterior and posterior aortic cusps in early systole, and the cross-sectional area is equal to the product of square *D* by 3.14 divided by 4 (Desai & Garry, 2018).

VTI can be estimated by obtaining the apical four-chamber (A4C) view, then tilting the ultrasound beams anteriorly to get the apical five-chamber (A5C) view, where the proximal ascending aorta, aortic valve, and left ventricular outflow tract (LVOT) come in the view (Matta et al., 2019). The pulse-wave (PW) Doppler is positioned within 15° to the LVOT, just in proximity to the aortic valve for the best flow estimation (Miller & Mandeville, 2016).

We calculated CI using CO, BW, and height, and we defined patients as “volume responders” if they had a ≥ 15% increase in the CI after the PLR test, and “non-responders” if they had no change or a change of < 15%.

The PVI was automatically calculated and recorded using an adhesive oximeter probe (LNCS Adtx, Masimo, Irvine, CA) placed on the finger and wrapped with a black protector for minimizing light interference and then connected to a Masimo Radical-7 monitor (Masimo Corp., Irvine, CA).

The dIVC is the percentage of variation in the IVC diameter during inspiration vs expiration. Which we measured by another single operator simultaneously, using the convex probe of ultrasound (Mindray M5, UMT-200/China) by the subcostal view approach using M-mode during the same respiratory cycle, then we calculated dIVC percentage by the following formula:

$$ \mathrm{Distensibility}\ \mathrm{index}=\frac{\mathrm{IVC}\ \max -\mathrm{IVC}\ \min }{\mathrm{IVC}\ \min}\times 100\%. $$

### Statistical analysis

Data were collected, revised, coded, and entered to the MedCalc software (ver. 19.1.0; MedCalc Software, Ostend, Belgium), and the Statistical Package for Social Sciences, version 25.0 (SPSS Inc., Chicago, IL, USA). The quantitative data were presented as mean, standard deviations, and ranges. Also, qualitative variables were presented as number and percentages.

The paired Student *t* test was used to compare between hemodynamic data before and after PLR. Independent sample *t* test was used to compare between responders and non-responders for normally distributed parametric variables, while for the non-parametric data, we used the Mann-Whitney test.

The receiver operating characteristic (ROC) curves and area under the curve (AUC; with 95% confidence intervals [CIs]) of PVI, dIVC, and CVP were calculated and compared for the assessment of the ability of these methods to predict fluid responsiveness.

The confidence interval was set to 95%, and the margin of error accepted was set to 5%. So, a *P* value < 0.05 was considered significant, a *P* value < 0.001 was considered as highly significant, and a *P* value > 0.05 was considered insignificant.

### Sample size

The PASS program is used, setting alpha error at 5% and power at 80%. The result from the previous study by Pişkin and Öz (Pişkin & Öz, 2017) showed that the AUC for diagnostic accuracy for PVI was 0.939, while for the diagnostic accuracy for IVC, the AUC was 0.79 in the study done by Long et al. (Long et al., 2017) Based on this, the needed sample is 40 responders and 40 non-responders.

## Results

After the exclusion of 24 patients due to exclusion criteria, mainly poor echogenicity (18 patients), the study was conducted on 88 patients; we divided them into two groups (48 responders and 40 non-responders). There were no significant differences in patient demographics between the groups as shown in Table 1.

**Table 1:** Patient demographics

Variables	Responders (***n*** = 48)	Non-responders (***n*** = 40)	***P***
**Age, mean ± SD, years**	57.30 ± 14.50	55.68 ± 14.61	0.655
**Male,** ***n*** **(%)**	25/48 (52.1%)	22/40 (55%)	0.790
**Height, mean ± SD, cm**	169.02 ± 9.30	167.43 ± 9.29	0.425
**Weight, mean ± SD, kg**	81.40 ± 8.24	80.13 ± 9.89	0.513
**BMI, mean ± SD, kg/m**^**2**^	28.00 ± 2.69	27.60 ± 3.59	0.544
**Body surface area, mean ± SD, m**^**2**^	1.93 ± 0.13	1.92 ± 0.15	0.630
**APACHE II**	24.71 ± 6.02	26.23 ± 4.60	0.195

Values are expressed as mean ± SD

*SD* standard deviation, *BMI* body mass index, *APACHE II* Acute Physiology and Chronic Health Evaluation

Hemodynamic data presented in Table 2 show a significant difference after PLR in the responders group (*P* < 0.5), while only HR showed a significant difference after PLR in the non-responders group. The baseline MAP, CVP, and CI of the responders were significantly lower than the non-responders (*P* < .05); the baseline PVI and dIVC were significantly higher than responders (*P* < 0.5), while HR readings showed no significant difference (*P* > .05) between the baselines of the two groups.

**Table 2:** Hemodynamic variables before and after passive leg raising

Variables	Responders (***n*** = 48)		Non-responders (***n*** = 40)	
	Before PLR	After PLR	Before PLR	After PLR
**HR, mean ± SD, beats/min**	93.81 ± 8.59	91.17 ± 8.18*	91.50 ± 6.42	90.58 ± 6.23*
**MAP, mean ± SD, mmHg**	62.56 ± 5.17	67.85 ± 5.16*	69.30 ± 5.20**	72.00 ± 5.79
**CVP, mean ± SD, mmHg**	4.20 ± 2.27	6.50 ± 2.25*	5.15 ± 2.33**	7.95 ± 2.41
**CI, mean ± SD, L/min/m**^**2**^	2.76 ± 0.20	3.22 ± 0.29*	3.20 ± 0.20**	3.53 ± 0.21
**PVI, mean ± SD (%)**	17.85 ± 2.58	11.77 ± 2.04*	11.65 ± 2.39**	8.05 ± 1.78
**dIVC, mean ± SD (%)**	26.14 ± 7.22	16.08 ± 4.99*	15.60 ± 5.12**	9.93 ± 4.44

Values are expressed as mean ± SD

*CI* cardiac index, *CVP* central venous pressure, *dIVC* caval index, *HR* heart rate, *MAP* mean arterial pressure, *PVI* pleth variability index, *SD* standard deviation

*P < 0.05 vs baseline

**P < 0.05 vs “responders”

The difference between CVP, PVI, and dIVC regarding performance is presented in Table 3. CVP at a threshold value ≤ 5 mmHg had 70.83% sensitivity and 47.50% specificity, with AUC = 0.612 (0.502–0.714), and was not significant (*P* = 0.0648). dIVC had 79.17% sensitivity and 80% specificity at a threshold value of > 19.42% with AUC = 0.886 (0.801–0.944) and was highly significant (*P* < 0.0001).

**Table 3:** The comparison of performances between parameters

Parameters	CVP	PVI	dIVC
**Threshold values**	≥ 5 mmHg	> 14%	> 19.42%
**Sensitivity (%)**	70.83	93.75	79.17
**Specificity (%)**	47.50	87.50	80
**AUC (95% CI)**	0.612 (0.502–0.714)	0.955 (0.889–0.988)	0.886 (0.801–0.944)
**LR+**	1.35	7.50	3.96
**LR−**	0.61	0.071	0.26
**PPV**	61.82	90	82.61
**NPV**	57.58	92.12	76.19
***P***	0.0648	< 0.0001	< 0.0001

*AUC (95% CI)* area under ROC curve (95% CI), *CVP* central venous pressure, *dIVC* caval index, *LR* likelihood ratio, *NPV* negative predictive value, *PPV* positive predictive value, *PVI* pleth variability index

**Fig. 1:**
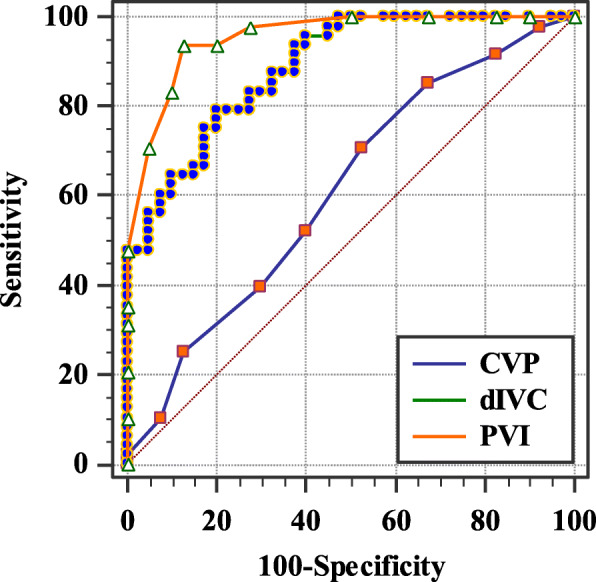
The receiver-operating characteristic curves comparing the ability of CVP, dIVC, and PVI to predict fluid responsiveness. CVP, central venous pressure; dIVC, caval index; PVI, pleth variability index

PVI had a 93.75 sensitivity and an 87.50 specificity at a threshold value of > 14% with AUC = 0.955 (0.889–0.988) and was highly significant (*P* < 0.0001). Figure 1 shows the ROC curves of the three methods comparing their effectiveness in the prediction of fluid responsiveness.

## Discussion

This observational cross-sectional study was done for 1 year in the surgical ICU. Collected data from 88 patients were analyzed for reaching the final results. We compared between PVI and dIVC regarding their reliability and effectiveness as simple non-invasive dynamic techniques in mechanically ventilated sedated patients with sinus rhythm.

The best way to test the sensitivity of the heart to preload changes is to detect the change in CO after a fluid bolus (Pierrakos et al., 2012). The usual fluid challenge has two concerns. First, one is that we should not use a procedure, which may entangle in itself harm and volume overload due to irreversibility (Muller et al., 2011). Therefore, we used PLR as a dependable test for the recognition of preload responsiveness (Monnet et al., 2016**)**.

The second concern is that we need to measure CO directly, so we used TTE as it is considered the gold standard dynamic technique in ICU (Desai & Garry, 2018), Moreover, a meta-analysis in 2016 revealed that it could predict fluid responsiveness accurately by PLR in ICU (Xiang et al., 2016). Patients in our study were defined as responders when their CO increased by 15% or more after PLR. The responders’ fraction for PLR in our work was 54.55%.

The deleterious effect of over fluid transfusion on different organs made a continuous need for new dynamic indicators for fluid management which are more preferable than unreliable static ones (Sakr et al., 2017). That was obvious in our study as CVP was a weak predictor for fluid responsiveness with best threshold value ≥ 5 mmHg and AUC of 0.612 (0.502 to 0.714).

Dynamic methods in fluid responsiveness identification are more accurate (Theerawit et al., 2016; Guérin et al., 2013)**,** but some of them require invasive procedures like arterial line insertion, and also, not all of them can provide continuous readings. Minimally or non-invasive cardiac output monitors have the least prerequisites and can be used in a variety of critically ill patients for estimating CO rapidly (Jalil & Cavallazzi, 2018) to minimize the risk (Scheer et al., 2002) to already ill patients.

PVI differs from other invasive dynamic techniques in providing the physicians with a numerical value continuously, automatically, and non-invasively (Sandroni et al., 2012). In a study done by Forget and others, they found that PVI-based goal-directed fluid therapy reduced the volume of infused fluids intraoperatively, so reduced lactate levels intraoperative and postoperative (Forget et al., 2010). It is suitable only for patients on mechanical ventilation (Keller et al., 2008), and it is not suitable for patients with open chest, irregular heart rhythm, RV failure, or high intra-abdominal pressure (Cannesson et al., 2011).

Different other factors can affect the accuracy of PVI as the anatomical region where we made our readings. The fingertips may be affected more than the cephalic region (Desgranges et al., 2011) (forehead and earlobe); also, vasoactive drugs such as norepinephrine, perfusion problems, or hypothermia may affect the vasomotor tone and decrease peripheral perfusion (Broch et al., 2011; Yamaura et al., 2007; Monnet et al., 2013).

Our observational study included only patients on mechanical ventilation with the exclusion of any patient with factors affecting the heart-lung interaction or with vasoactive drug infusion, so our study results are not affected by these factors. On the other hand, we used fingertip measurements as an easier way to calculate PVI for fluid responsiveness identification.

The ROC analysis in our study revealed that the best PVI threshold value was more than 14%, with 93.75% sensitivity, 87.50% specificity, and AUC (95% CI) for the ROC curve of 0.955 (0.889–0.988).

In a recent meta-analysis done by Liu et al. which was including 25 studies, they concluded that PVI has a good bedside reliability in ICU especially with 79% sensitivity, 88% specificity, and AUC of 0.89 (0.86–0.92) also its limited ability in the prediction of fluid responsiveness in general (Liu et al, 2019). However, Chu et al.’s meta-analysis showed higher sensitivity of fluid responsiveness in operating theaters than ICU but with the same specificity and nearly equal AUC of 0.89 (0.85–0.92) in operating theater (OR) and AUC of 0.90 (0.82–0.94) in ICU (Chu et al., 2016). This is in line with our results; also, our study was in a surgical ICU using PLR with the exclusion of any patient on vasoactive drugs, which was not met with the two included meta-analysis studies.

Lately, ultrasound became one of the most important devices in ICU; it can be used as a bedside non-invasive method for the assessment of fluid responsiveness in daily ICU practice (Theerawit et al., 2016; Orso et al., 2016) by dIVC using variation in the diameter of the IVC (Barbier et al., 2004).

In a systematic review and meta-analysis done by Long et al. (Long et al., 2017), they found that pooled results of dIVC in mechanically ventilated patients had AUC of 0.79 with a sensitivity of 67% and a specificity of 68%, which is not in line with our results.

In another recent systematic review and meta-analysis done by Huang et al. (Huang et al., 2018), dIVC has better performance in mechanically ventilated shocked patients with a pooled AUC of 0.82 (95% CI 0.79–0.85) with a specificity of 80% and a sensitivity of 69%, which is near to our study results with 79.17% sensitivity and 80% specificity at a threshold value of > 19.42% with AUC = 0.886 (0.801−0.944).

However, several studies showed poor performance of dIVC. A study included 44 medical and surgical septic mechanically ventilated patients who had an AUC of 0.43 and 95% CI 0.25–0.61) with 38% sensitivity and 61% specificity (Charbonneau et al., 2014).

Many factors can affect dIVC measurements and cause this difference between studies as respiratory compliance (Lakhal et al., 2011), difference in adjusted tidal volume, amount and type of infused fluids (Barbier et al., 2004), and factors affecting intra-abdominal pressure (Bendjelid & Romand, 2012; Santa-Teresa et al., 2012).

The results of our study showed that assessment of PVI and dIVC non-invasively were good predictors for fluid management and responsiveness prediction using PLR technique in the surgical ICU mechanically ventilated patients.

### Limitations

Our observational study has many limitations. The first one was in the accuracy of measuring CO by VTI using TTE. It necessitates good echogenicity of the patients, so we excluded 18 patients not fulfilling these criteria before data analysis.

The second limitation was in the comparison between PVI and dIVC; it is difficult to equalize blood volume added to circulation to all patients after PLR. However, the reversible PLR technique was proven trustworthy for preload responder identification (Monnet et al., 2016) with less harm to the candidate patient than irreversible transfusing of the same volume of crystalloid or colloid to all patients including the non-responders with no benefit.

The third limitation was intra-abdominal pressure, as we did not measure it in our patients. However, we excluded all patients with any expected cause of increased intra-abdominal pressure like ascites, pregnancy, abdominal malignancy, distension, and acute abdomen.

## Conclusion

PVI and dIVC can be used in the assessment of fluid responsiveness of the intubated ventilated sedated patients with sinus rhythm in ICU, and both methods are non-invasive and can be performed at the bedside, but PVI has the advantage of being continuous, operator-independent, and more reliable than dIVC.

## Data Availability

The datasets used and/or analyzed during the current study are available from the corresponding author on reasonable request.

## Abbreviations

APACHE II: Acute Physiology and Chronic Health Evaluation
AUC: Area under the curve
BMI: Body mass index
BP: Blood pressure
CI: Cardiac index
CO: Cardiac output
CVP: Central venous pressure
*D*: Diameter
dIVC: Inferior vena cava distensibility index
HR: Heart rate
ICU: Intensive care unit
ITP: Intrathoracic pressure
IV: Intravenous
IVC: Inferior vena cava
LVOT: Left ventricular outflow tract
MAP: Mean arterial pressure
OR: Operating theater
PAOP: Pulmonary artery occlusion pressure
PLR: Passive leg raising
PPV: Pulse pressure variation
PVI: Pleth variability index
PW: Pulse-wave
SV: Stroke volume
SSV: Stroke volume variation
TTE: Transthoracic echocardiography
VR: Venous return
VTI: Velocity time integral
